# Emotion Forecasting: A Transformer-Based Approach

**DOI:** 10.2196/63962

**Published:** 2025-03-18

**Authors:** Leire Paz-Arbaizar, Jorge Lopez-Castroman, Antonio Artés-Rodríguez, Pablo M Olmos, David Ramírez

**Affiliations:** 1 Signal Theory and Communications Department Universidad Carlos III de Madrid Leganés Spain; 2 Department of Psychiatry, Public Health, Radiology, Nursing and Medicine University of Santiago de Compostela Santiago de Compostela Spain; 3 Centro de Investigación Biomédica en Red de Salud Mental Madrid Spain; 4 Department of Adult Psychiatry Nimes University Hospital Nimes France; 5 Instituto de Investigacion Sanitaria Gregorio Marañón Madrid Spain; 6 Evidence-Based Behavior (eB2) Leganés Spain

**Keywords:** affect, emotional valence, machine learning, mental disorder, monitoring, mood, passive data, Patient Health Questionnaire-9, PHQ-9, psychological distress, time-series forecasting

## Abstract

**Background:**

Monitoring the emotional states of patients with psychiatric problems has always been challenging due to the noncontinuous nature of clinical assessments, the effect of the health care environment, and the inherent subjectivity of evaluation instruments. However, mental states in psychiatric disorders exhibit substantial variability over time, making real-time monitoring crucial for preventing risky situations and ensuring appropriate treatment.

**Objective:**

This study aimed to leverage new technologies and deep learning techniques to enable more objective, real-time monitoring of patients. This was achieved by passively monitoring variables such as step count, patient location, and sleep patterns using mobile devices. We aimed to predict patient self-reports and detect sudden variations in their emotional valence, identifying situations that may require clinical intervention.

**Methods:**

Data for this project were collected using the Evidence-Based Behavior (eB2) app, which records both passive and self-reported variables daily. Passive data refer to behavioral information gathered via the eB2 app through sensors embedded in mobile devices and wearables. These data were obtained from studies conducted in collaboration with hospitals and clinics that used eB2. We used hidden Markov models (HMMs) to address missing data and transformer deep neural networks for time-series forecasting. Finally, classification algorithms were applied to predict several variables, including emotional state and responses to the Patient Health Questionnaire-9.

**Results:**

Through real-time patient monitoring, we demonstrated the ability to accurately predict patients’ emotional states and anticipate changes over time. Specifically, our approach achieved high accuracy (0.93) and a receiver operating characteristic (ROC) area under the curve (AUC) of 0.98 for emotional valence classification. For predicting emotional state changes 1 day in advance, we obtained an ROC AUC of 0.87. Furthermore, we demonstrated the feasibility of forecasting responses to the Patient Health Questionnaire-9, with particularly strong performance for certain questions. For example, in question 9, related to suicidal ideation, our model achieved an accuracy of 0.9 and an ROC AUC of 0.77 for predicting the next day’s response. Moreover, we illustrated the enhanced stability of multivariate time-series forecasting when HMM preprocessing was combined with a transformer model, as opposed to other time-series forecasting methods, such as recurrent neural networks or long short-term memory cells.

**Conclusions:**

The stability of multivariate time-series forecasting improved when HMM preprocessing was combined with a transformer model, as opposed to other time-series forecasting methods (eg, recurrent neural network and long short-term memory), leveraging the attention mechanisms to capture longer time dependencies and gain interpretability. We showed the potential to assess the emotional state of a patient and the scores of psychiatric questionnaires from passive variables in advance. This allows real-time monitoring of patients and hence better risk detection and treatment adjustment.

## Introduction

### Background

The presence of a specific mood status is a necessary criterion for many psychiatric diagnoses, as outlined in the *Diagnostic and Statistical Manual of Mental Disorders* [1], and self-perceived mood is a fundamental component of assessing mental states in psychiatry [2,3]. Therefore, precise monitoring and following of mood conditions play a vital role in mental health care. For instance, both positive and negative mood states, as well as their fluctuations, have demonstrated their predictive value for significant outcomes, such as compulsive overeating episodes in bulimia nervosa, adherence to treatment in bipolar disorder, and opioid use disorders [4,5]. In recent years, technological advancements have facilitated the real-time tracking of individuals’ self-reported mood status. One notable advancement is the use of smartphone-delivered ecological momentary assessment (EMA), which allows for the analysis of an individual’s experiences, behavior, and emotions as they unfold in their natural environments [6]. However, the effectiveness of this method of mood state evaluation largely depends on the individual’s level of self-awareness and ability to interact with the EMA platform. In many cases, psychiatric disorders can cause behavioral changes that decrease the likelihood of individuals engaging with an EMA tool, resulting in missing data. Consequently, a crucial research priority is the development of objective behavioral biomarkers for mood states that can be passively sensed without requiring active involvement from individuals.

By harnessing the power of patients’ mobile phones and wearable devices, it has become feasible to gather continuous sensor data in a noninvasive manner, providing valuable insights into their daily activity patterns [7]. These models hold the potential to forecast mental health crises and detect abnormal behavioral patterns, facilitating early intervention [8].

In this study, we examined daily summaries of passively collected behavioral data, treating them as multivariate time series. Passively collected data refer to behavioral information recorded through sensors embedded in devices such as wearables and mobile phones, where patient interaction is not required for data capture. This passive data collection is supplemented by actively provided inputs from patients, such as self-reported emotions or responses to questionnaires tailored to the study context. The dataset used in this research was obtained using the Evidence-Based Behavior (eB2) app, a platform developed by our research group and implemented across multiple studies. These studies encompass a variety of patient cohorts, providing extensive behavioral data complemented by responses to psychological, quality-of-life, and nutrition-related questionnaires, depending on the specific objectives of each study.

For time-series analysis, we used transformer models, which are particularly well suited for capturing long-range dependencies within sequential data [9]. Leveraging the advantages offered by transformer models for time-series analysis, we aimed to uncover underlying patterns in the data collected over time. The attention mechanism in transformers is a fundamental feature that enables the model to prioritize relevant temporal dependencies within the input sequence. By assigning varying weights to different time samples in the sequence, transformers can effectively capture and integrate the most pertinent information for accurate forecasting [10,11]. Furthermore, transformer models exhibit the ability to handle sequences of variable lengths. This flexibility renders transformers scalable to datasets containing many time points, accommodating diverse time-series lengths without sacrificing performance [12]. These attributes position transformers as a promising option for time-series forecasting, as they facilitate the modeling of complex temporal patterns and enhance forecasting accuracy.

### Related Work

#### Overview

This section covers different aspects related to depression diagnosis and tracking, including research in patient emotional state monitoring and the reliability of the Patient Health Questionnaire-9 (PHQ-9) for diagnosis. Moreover, it explores how researchers are using passive observations to obtain standardized and objective insights into patients’ self-reported states. Following this, we introduce works that apply machine learning and deep learning methods to analyze such data for enhanced diagnosis accuracy and longitudinal patient tracking. Finally, we provide some background on transformer-type attention models for time-series forecasting.

#### Depression Diagnosis and Tracking

As numerous studies have shown [13-15], depression remains the worldwide leading cause of disability. However, conventional diagnosis and tracking approaches primarily rely on self-reported depressive symptoms in clinical settings—methodologies that were established over half a century ago. These methods typically entail survey completion or face-to-face interviews, offering limited accuracy, ecological validity, and reliability while imposing significant costs for monitoring and scalability [16]. Moreover, the subjective nature of patient and clinician evaluations, combined with the fluctuating nature of mental health conditions over time, emphasizes the need for ongoing, longitudinal assessments to accurately capture these nuances effectively. In terms of assessing depression, the PHQ-9 has shown to be reliable for the criteria-based diagnosis of this disorder, alongside giving a valid measure of depression severity. These characteristics, plus its brevity, make the PHQ-9 a useful clinical and research tool [17].

A set of studies focused on the relationship between mood variability and some psychiatric disorders. In this work, we used mood in the sense of the subjective variation of the patient’s emotional state, as expressed by the patient [18]. Research indicates that mood variability including hypomania, cyclothymia, and hyperthymia has been described in 40% to 50% of patients with depression and that such variability could also characterize anxiety disorders [19,20]. Over the past 2 decades, there has been a surge in research linking various patterns of short-term emotional change to adaptive or abnormal psychological functioning, often with conflicting results [21]. Psychiatric decompensations are characterized by specific patterns of emotional fluctuations across time and provide insight into what constitutes optimal and suboptimal emotional functioning.

#### Advances in EMA and Passive Monitoring

To prevent bias and capture changes in behavior over time and across different contexts, several researchers proposed a shift from relying solely on global retrospective self-reports collected during research or clinic visits in clinical psychology assessment [22]. In EMA, data are repeatedly collected on an individual’s current behaviors and experiences as they happen in their everyday environments [6,23]. EMA helps reduce memory bias, provides more accurate insights into daily life, and allows for the study of small-scale influences on behavior in real-world settings. Technologies used for EMA range from traditional written diaries and phone calls to electronic diaries and physiological sensors [22]. In this case, an EMA-style monitoring is conducted to rely solely on passively collected variables through mobile phone sensors and wearables, excluding all the patient-reported data.

Several studies that reviewed the passive follow-up of patients with different conditions, including bipolar disorder, schizophrenia, and depression, highlighted the potential of passive biomarkers for the monitoring of different types of disorders. Particularly, variables such as accelerometry, location, audio, and use data showed a high general performance [24]. Other studies explored the detection of daily-life behavioral markers through mobile phone GPS and use sensors. They showed that features extracted from these sensors provided markers strongly related to depressive symptom severity [21]. In this line, one of the variables that has shown a correlation with the individual’s mental state is daily activity [25,26]. A study focused on detecting emotional state instabilities through passive data found that 3 weeks of continuous, passive recordings were enough to reliably predict mood changes, obtaining average and median errors of mood instability scores within a margin of 5% [27].

#### Machine Learning and Deep Learning Approaches for Behavioral Data Analysis

Due to the potential demonstrated by these variables in predicting emotional states and their variability in patients, previous works have focused on the application of machine learning and deep learning algorithms to analyze these data [28]. A study by Ghandeharioun et al [13] applied machine learning methods to data on sleep behavior, motion, phone-based communication, location changes, and phone use patterns to impute missing clinical scores from self-reported measures and predict depression severity from these continuous sensor measurements. Similarly, other studies evaluated the performance of random forest and support vector machine classifiers for binary classification of the PHQ-9 score, resulting in 60.1% and 59.1% accuracy, respectively, demonstrating a proof of concept for the detection of depression from passive features [29]. Following this approach, diagnostic meta-analyses have demonstrated the effectiveness of the PHQ-9 for depression screening using mobile devices through various machine learning techniques [30].

Recent studies [30,31] have emphasized the importance of continuous follow-up and the analysis of temporal behavioral patterns in the longitudinal monitoring of patients. Aiming to address the lack of clarity on the temporal scale, specificity, and person-specific nature of the associations between smartphone data and affective symptoms, a study was conducted on smartphone-based passive sensing to identify within- and between-person digital markers of depression and anxiety symptoms over time [31]. Here, hierarchical linear regression models and temporal windows were used to understand the timescale at which sensed features relate to mental health symptoms and explore the predictions in the distal, medial, and proximal times. In line with this, other studies used multilevel modeling to examine the relationships between daily mood and mood variability with symptoms of depression, generalized anxiety, and social anxiety to confirm the empirical evidence linking EMA of mood variability with psychiatric disorders [32]. The findings showed both common and specific emotional dynamics that defined the severity of affective symptoms.

Our research aligned with previous studies by aiming to predict self-reported emotional states and their fluctuations as well as the PHQ-9 scores [33]. However, our methodology differs from conventional approaches by integrating into patients’ natural daily routines rather than relying on an experimental setup. Besides, our sample included patients with a variety of disorders who were expected, but not compelled, to actively report data via the app. This setup introduced challenges, notably the substantial presence of missing data, which we addressed using a hidden Markov model (HMM), as documented in previous literature [34]. We build upon this paper, which predicted emotional valence from passive variables, using HMM to handle missing values and classification methods. With the proposed model, we enhanced the emotional valence prediction achieved in this paper and obtained a more reliable prediction as the time horizon expanded. In addition, we included the prediction of the scores of the PHQ-9 questions.

To leverage the continuous acquisition of passive variables and work with these temporal data sequences, this study adopted a transformer-based approach. Transformer models, based on attention mechanisms, offer several advantages for time-series forecasting. The transformer architecture, as introduced by Vaswani et al [10], excels at capturing long-range dependencies, making it well suited for time-series data where distant historical information can be crucial. Attention mechanisms within transformers enable contextual understanding, allowing the model to weigh the significance of past elements, thereby improving forecasting accuracy [10,12].

In addition to the previously mentioned studies, several publications delved into the application of transformers to multivariate time-series data [12,35,36], showcasing the flexibility and adaptability of the transformer architecture in capturing complex temporal relationships. Initially developed for natural language processing tasks, transformers have demonstrated a seamless transition to time-series forecasting due to their inherent capability to model temporal data. This shift underscores the versatility and robustness of transformer-based models in addressing diverse sequential data tasks beyond language processing. Furthermore, pretrained transformer models can be fine-tuned for specific forecasting tasks, leveraging insights from diverse datasets, as demonstrated in various transfer learning applications. Finally, attention weights also contribute to their utility in analyzing and forecasting time-series data in a more interpretable way [37].

To the best of our knowledge, no previous studies have used transformer models specifically for emotion recognition relying solely on behavioral (passive) data from mobile devices. This represents a novel direction in leveraging behavioral data for emotion classification and change detection, particularly in real-world, noninvasive contexts.

In other domains, emotion recognition has been explored using transformer models applied to various data modalities. For text-based emotion recognition, transformer models such as Bidirectional Encoder Representations from Transformers (BERT) and GPT proved to be effective in capturing emotional nuances in textual data. For instance, Xie et al. [38] used GPT (developed by OpenAI) to encode dialogue features, while Li et al. [39] combined Robustly Optimized BERT Pretraining Approach (RoBERTa, developed by Facebook AI) embeddings with wav2vec (version 2.0, developed by Facebook AI) for cross-modal emotion recognition.

For audio-based emotion recognition, speech data have been modeled using architectures such as wav2vec (version 2.0). Chen and Rudnicky [40] fine-tuned wav2vec (version 2.0) for detailed speech emotion analysis, while Sun et al [41] combined it with BERT for multimodal integration.

Transformers have also been used for visual-based emotion recognition, where facial expressions and gestures were analyzed. Praveen et al [42] used the multihead attention mechanism to fuse visual and audio features, effectively capturing both spatial and temporal dynamics.

Multimodal emotion recognition, which combines text, audio, and visual data, has been a common approach. Xie et al [38] proposed a Crossmodality Fusion Transformer, and Zhao et al [43] introduced MEmoBERT for cross-modal emotion classification tasks.

In the field of physiological emotion recognition, transformer-based models such as modality-agnostic transformer based self-supervised learning [44] and conformer [45] have demonstrated high accuracy in analyzing electroencephalogram and electrocardiogram signals for emotion classification.

In contrast to these approaches, our work focused exclusively on behavioral data collected passively through smartphones and wearable devices, without relying on more invasive techniques such as video, voice, or physiological signals such as electroencephalogram. This distinction offered significant advantages. By aligning with natural patient behavior and environments, our method reduced intrusiveness, ensured scalability, and facilitated seamless integration into daily life, making it particularly suitable for real-world applications.

### Objectives

The general goal was to acquire objective indicators of patients’ conditions and fluctuations in their emotional well-being from passive biomarkers. For this, we focused on predicting the emotional valence of patients as well as the PHQ-9 score. This approach aimed to address the challenge of subjectivity and the absence of continuous monitoring in psychiatry, ultimately aiding in the identification of potentially risky situations. This would facilitate timely intervention and treatment adaptation, thus improving the quality of life of the patients and their environment. We strived to achieve this prediction several days in advance of the actual event. Moreover, the incorporation of attention mechanisms served the additional purpose of advancing our understanding of behavioral patterns.

## Methods

### Recruitment: Patient Inclusion and Exclusion Criteria and Indications

Participants were eligible for inclusion in the study if they were aged ≥18 years and were diagnosed as clinical outpatients with mental disorders by specialists or if they were attending therapy groups. Among these groups, there were 3 main categories: high suicidal risk, eating disorder, and common mental disorder (CMD). CMD encompasses a group of distress states manifesting with anxiety, depression, and unexplained somatic symptoms that are typically encountered in community and primary care settings [46]. Furthermore, patients from other studies, including cohorts of patients affected by cancer, HIV, obstructive sleep apnea, and cardiac conditions, as well as control patients, were included.

Participants were required to own a smartphone running on Android or iOS operating systems, which they connected to a Wi-Fi network at least once per week. Only participants who provided written informed consent for the eB2 study were included.

Patients received instructions from the clinicians at the beginning of follow-up. For most cohorts, only information regarding the app’s functionality was provided. However, 2 groups received specific guidance: patients with eating disorders were required to compulsorily complete meal entries, while patients with CMD were encouraged to regularly log their emotions and periodically complete the PHQ-9. There was no obligation or a set number of required responses for these tasks.

Passive data sources included both mobile and wearable devices. In some studies involving patients with HIV, cancer, or obstructive sleep apnea, patients were provided with wearables. For the remaining studies, if patients had their own wearable, information was extracted from the most reliable data source.

### Data

This study was conducted on a sample of 4403 patients from 8 distinct cohorts, each characterized by a different pathology or condition. Out of the 4358 patients, the patient cohorts included individuals with CMD (n=1785, 40.54%), eating disorder (n=1477, 33.54%), high suicidal risk (n=413, 9.38%), cancer (n=84, 1.91%), obstructive sleep apnea (n=48, 1.09%), HIV (n=24, 0.54%), cardiology-related conditions (n=20, 0.45%), as well as control participants (n=507, 11.51%).

The data for this study were derived from secondary analyses of multiple clinical studies conducted in collaboration with various hospitals. All studies were designed to monitor patients with distinct health conditions using the eB2 platform. Despite differences in data sources, standardized protocols for data collection were applied across all studies to ensure consistency in data quality. All users were Spanish or French. Among them, 57.48% (2531/4403) were female, 40.93% (1802/4403) were male, and sex information was missing for the remaining 1.59% (70/4403) of the users. All age groups were well represented, with a mean age of 46 (SD 14.8; range 18-77) years at the start of the measurement period.

Patient monitoring was conducted using the eB2 MindCare app [47,48]. The eB2 app operates by harnessing information from diverse sources within the patient’s ecosystem. Using phone sensors, Google Fit (Google LLC), and wearable devices, it acquires data at varying intervals, facilitating a nuanced understanding of the patient’s daily activities. In parallel with this passive monitoring, patients had the option to input subjective experiences, sleep patterns, and emotional states throughout the day. Emotional states were cataloged within the app, offering 20 options in a spectrum from anger to delight [49,50].

Daily summaries of these data were the primary focus, although alternative granularities were also considered (such as hours and minutes). Therefore, when predicting sequences or emotions at the next temporal moment, this temporal interval was in days. The data collection period for this study spanned 8 years, from 2016 to 2023, and the average duration of passive activity sequences for patients was 224 days, with an SD of 200 days.

The data used as inputs for training the models were daily summaries of the following passive variables: step count, covered distance, sleep hours, app use, time at home, number of visited places, and practiced sport (a binary variable indicating whether the patient practiced sports during the day). The targets we aimed to predict from these passive data were patients’ emotional valence and PHQ-9 scores. The ground truth values for these targets were derived from self-reported emotions and questionnaire answers that patients completed through the app. A more detailed description of the different cohorts is included in Multimedia Appendix 1.

### Preprocessing

To ensure the variables were sampled at the same frequency, daily summaries were created for each variable. These daily summaries were derived by aggregating the data according to the specific variable.

Sleep hours, distance, steps, and app use were calculated using passive data collection methods from various sources. Sleep hours were determined based on a prioritization hierarchy: user-entered data were the most prioritized, followed by wearable devices; mobile data; and finally, a sleep estimation model. Daily distance was calculated using GPS signals from mobile devices, collected every 5 minutes. Step counts were gathered at intervals of 1 to 5 minutes, depending on the manufacturers of the smartwatch devices. To calculate daily totals, step data from each slot were merged by priority (wearables over mobile). App use was recorded every 5 minutes, capturing which apps were used and the duration of their use. For these 4 variables, once the data were collected and selected from the priority sources, the data were summed to obtain the daily summary of each variable.

To identify home and work locations, the DenStream algorithm was used. Location data were collected over a 15-day period to form clusters. Once the clusters were established, incoming location data were tagged in real time as either home, work, or other. The cluster definitions were updated every 30 days following the initial 15-day period. On the basis of this clustering process, the time a user spent at home was calculated. Data were collected every 5 minutes and tagged upon entry to indicate the cluster corresponding to home. The total time at home was measured in seconds and aggregated for daily summaries.

The variable practiced sport was a Boolean value indicating whether a user engaged in physical activity lasting at least 15 minutes during the day. This variable was updated whenever new physical activity data were received. Physical activity detection was based on three primary sources: (1) activities automatically labeled by devices (eg, mobile phones and wearables), (2) manually logged activities by users through the provider’s app, and (3) activities logged directly into the eB2 MindCare platform.

**Figure 1:**
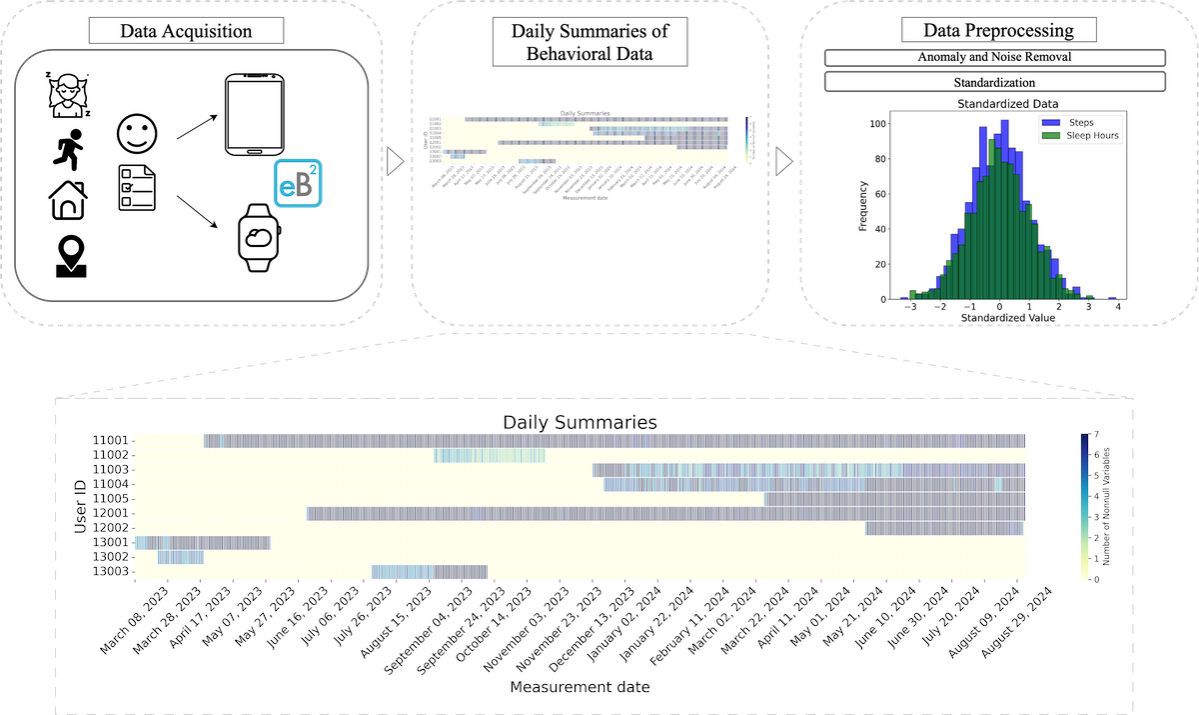
The data preprocessing pipeline: data acquisition, obtention of the daily summaries, and standardization of behavioral data. The sequence shown in the daily summaries displays the temporal sequence of passive data for 5 different patients. The intensity of the lines indicates the amount of nonmissing behavioral data the patient has for that day.

To address the variations between sensors and data formats, which resulted in anomalies and noise in the information, a preprocessing stage was implemented. This included removing negative values, thresholding the time-related variables to 24 hours, the time step count to 30,000 per day, and the distance to 500 km. Finally, data were standardized over all the patients’ sequences (mean 0, SD 1 for input features). Standardization was performed to ensure all variables had a uniform scale, which improved the efficiency and performance of machine learning algorithms. This was particularly important for models sensitive to differences in feature scales, such as neural networks or distance-based methods. Figure 1 shows the sequence of data preprocessing.

Regarding passive data, the mean percentage of missing data was approximately 60.23% (594,699/986,909), with step count (519,168/986,909, 52.61% of total missing) having the fewest missing values and time at home being the least complete (685,614/986,909, 69.47% total missing). Table 1 shows the percentage of missing values per passive variable grouped by year.

Regarding targets, Table 1 shows the percentage of missing emotion ratio and PHQ-9 answers in comparison with the passive data. It can be observed that the variables reflecting the patients’ mood (self-reported emotions and PHQ-9 answers), which they must enter actively, had the lowest percentage of registered data. This is why we aimed to predict these 2 variables using passive data, as they were more complete and allowed for continuous monitoring of the patient’s condition without relying on the patients to input the data.

In this study, we handled emotions following the classification scheme by Russell [51], which characterizes emotions in a 2D space. The circumplex model by Russell [52] proposed that emotions could be understood along 2 independent and bipolar dimensions: valence (pleasantness or unpleasantness) and activation (high arousal or low arousal). Independence implied that valence and activation were uncorrelated, while bipolarity suggested that opposite emotions lied at opposite poles of each dimension. For example, happiness and sadness represented opposite ends of the pleasantness spectrum, whereas emotions such as tension and sleepiness were at opposite extremes of the activation dimension.

Using this framework, emotions reported by patients were assigned a level of valence as positive (pleasant), negative (unpleasant), or neutral. For instance, positive valence emotions included happiness, enthusiasm, or satisfaction, while negative valence emotions included sadness, anger, or frustration. Neutral valence emotions fell in the center of the pleasantness spectrum, representing a lack of strong affective polarity [53]. Related studies [54] similarly relied on the model proposed by Russell [52] to categorize emotions, either in 1D using valence or in 2D with both valence and arousal. From this scheme, we focused on emotional valence, which was the first prediction target.

The daily valence was determined by the difference between the counts of positive and negative emotions and can take values between 0 and 2 (negative, neutral, and positive valence).

In addition, the PHQ-9, comprising the 9-item depression module extracted from the full Patient Health Questionnaire, was examined as another target variable. According to this questionnaire, diagnosis of major depression was established if “more than half the days” over the past 2 weeks exhibited the presence of ≥5 depressive symptom criteria, with one of these symptoms being either depressed mood or anhedonia [17]. The responses to the questionnaire were derived from the cohort of patients with CMD, comprising a total of 597 completed surveys. In the context of this investigation, each of the 9 questionnaire items was treated independently. This approach was adopted due to the diverse nature of the questions, which collectively encompassed various facets of the patients’ daily experiences. The simultaneous prediction of all scores posed a considerable methodological challenge in this initial endeavor.

**Table 1:** Percentage of missing daily data for each passive variable^a^ by year.

Year	Total number of records per year, n	Steps, n (%)	Distance, n (%)	Sleep, n (%)	App use, n (%)	Time at home, n (%)	Location clusters, n (%)	Emotions ratio, n (%)	PHQ-9^b^, n (%)
2016	9548	1243 (13.01)	9548 (100.00)	9511 (99.612)	9548 (100)	9548 (100)	9548 (100)	—^c^	—
2017	19,009	2122 (11.16)	19,009 (100)	18,938 (99.62)	19,009 (100)	19,009 (100)	19,009 (100)	—	—
2018	104,755	63,291 (60.42)	41,528 (39.64)	74,188 (70.82)	94,501 (90.21)	51,521 (49.18)	46,886 (44.76)	—	—
2019	183,919	92,552 (50.32)	72,906 (39.64)	137,889 (74.97)	144,283 (78.45)	93,272 (50.71)	88,998 (48.39)	—	—
2020	139,296	82,317 (59.10)	75,816 (54.43)	10884 (78.14)	105,514 (75.75)	91,684 (65.82)	89,711 (64.4)	—	—
2021	165,154	99,234 (60.09)	121,355 (73.48)	93,075 (56.36)	86,382 (52.3)	128,809 (77.99)	128,276 (77.67)	—	—
2022	239,773	119,631 (49.89)	189,051 (78.85)	128,221 (53.48)	139,454 (58.16)	192,852 (80.43)	191,660 (79.93)	—	—
2023	125,455	58,778 (46.85)	96,824 (77.18)	65,042 (51.84)	69,014 (55.01)	98,919 (78.85)	98,449 (78.47)	—	—
Total	986,909	519,168 (52.60)	626,037 (63.43)	635,705 (64.41)	667,705 (67.66)	685,614 (69.47)	672,537 (68.15)	950,833 (96.34)	967,171 (98.00)

^a^Passive variables include steps, distance, sleep, app use, time at home, location clusters, emotions ratio, and PHQ-9.

^b^PHQ-9: Patient Health Questionnaire-9.

^c^Not available.

Originally, this score comprised 4 classes. However, due to the insufficient number of answers for each type and the frequency-based nature of the responses (ranging from “not at all” to “nearly every day”), the intermediate classes sometimes merged with the 2 extreme groups. Consequently, we decided to classify the answers into 2 broader classes: 0 (representing “not at all” and “several days” as low frequency) and 1 (representing “more than half of the days” and “nearly every day” as high frequency).

### Models

#### Overview

**Figure 2:**
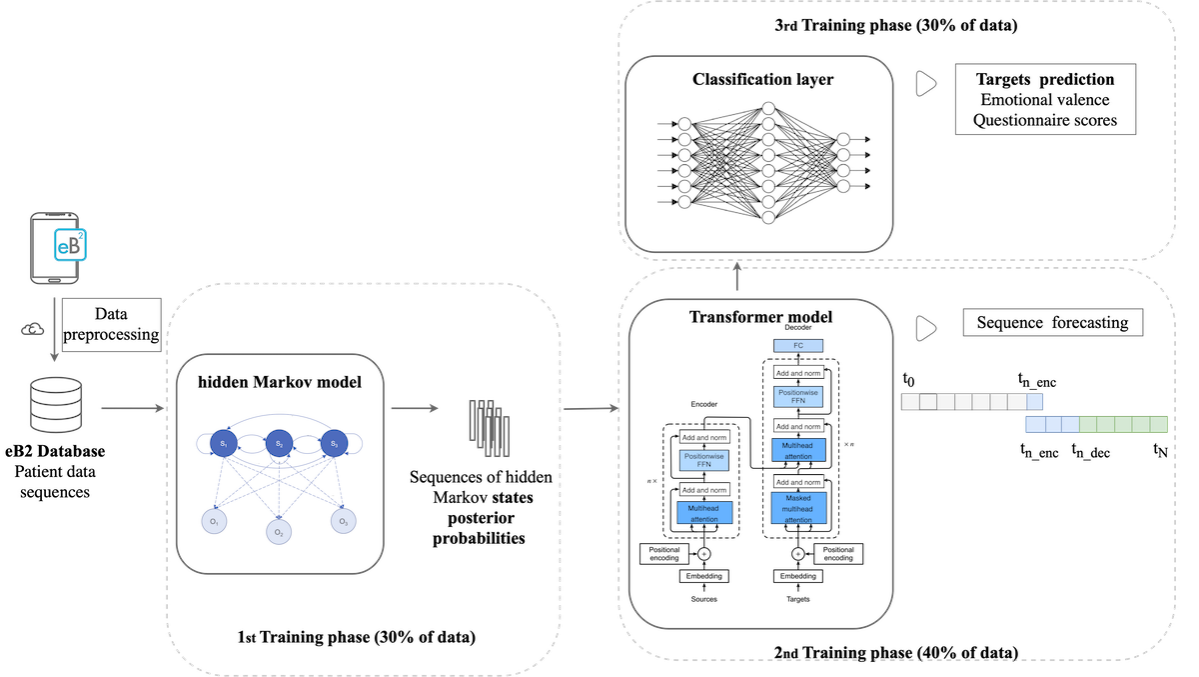
A graphical abstract of the proposed scheme illustrating the underlying architecture, comprising 3 primary blocks. The first block involves the use of hidden Markov models (HMMs) to address missing data and extract posterior state probabilities. The second block uses a transformer model equipped with an attention mechanism to facilitate pattern recognition and time-series forecasting. Finally, the scheme incorporates a classification layer responsible for patient-reported variables, namely, emotional valence or Patient Health Questionnaire-9 score. eB2: Evidence-Based Behavior; FFN: feed-forward network; t₀: initial time of the sequence; tₙ_enc: length of the sequence of observed data used as input for the encoder; the encoder is fed with observed data from t₀ to tₙ_enc, in this case, 30 days; tₙ_dec: length of the sequence of observed data used as input for the decoder; the decoder is fed with data from tₙ_enc to tₙ_dec, in this case, for training purposes, the observed sequence consisted of 20 days; tN: forecasting horizon in days, it does not have a fixed value, for example, in this study, N is either 1 or 3, depending on the prediction horizon.

Our global model, illustrated in Figure 2, consisted of 3 main submodels that were trained separately. Hence, we considered that the global model was trained in three stages: (1) training of the HMM to deal with missing fields, (2) the autoregressive transformer to pretrain the model to capture the temporal structure of the data, and (3) the classification model itself.

Initially, the raw data underwent the preprocessing and quality control mentioned previously. To prevent data leakage between the different training phases and biases, the original dataset was divided into 3 subsets for training and validation purposes, with 30% (296,072/986,909), 40% (394,765/986,909), and 30% (296,072/986,909) of the data allocated to the HMM, transformer, and classification layers, respectively, as shown in Figure 2. The partitions were made by assigning equitable percentages of each cohort to each subset to mitigate biases, assigning unique different users to each of them. The following sections describe the 3 main stages in detail.

#### Probabilistic Generative Model for Dealing With Missing Data: HMMs

Our objective was to evaluate the efficacy of transformer models in enhancing time-series forecasting. However, transformer models do not inherently accommodate missing data, presenting a challenge for our analysis. To address this limitation, we used HMMs. In addition, we leveraged the latent space representation provided by HMMs, using the posterior probabilities of the hidden states for training the transformer model.

HMMs, commonly used in time-series analysis, represent a temporal variant of Markov models [55,56]. These generative models are characterized by a collection of observable variables, and a notable advantage lies in their ability to manage missing data without necessitating prior imputation, achieved through marginalization. In the HMM, a sequence of observable variables O is generated by a corresponding sequence of internal hidden states S. However, these hidden states are not observed directly. Instead, transitions between hidden states follow the assumption of a first-order Markov chain. This transition process is defined by a start probability vector π and a transition probability matrix A. In addition, each observable emission is associated with a probability distribution, conditioned on the current hidden state. These emission probabilities are specified by parameter B. Together, these parameters λ=(π, A, B) fully define the HMM.

**Figure 3:**
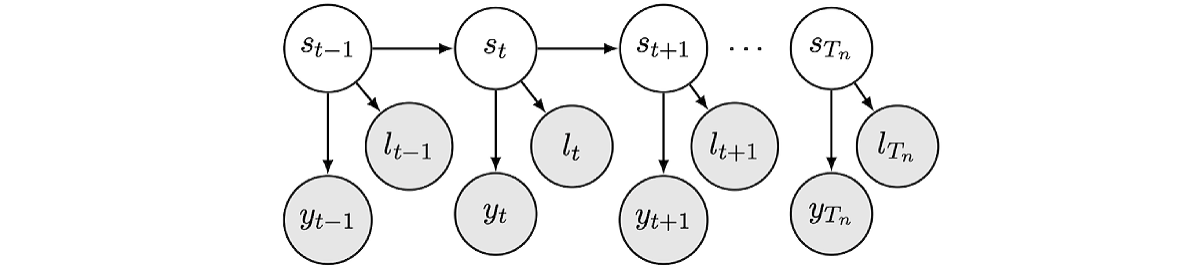
Architecture of the heterogeneous hidden Markov model. The model is described by their hidden states sequence (s0:Tn), continuous observations sequence (y0:Tn), and discrete observations sequence (l0:Tn). In our case, (l0:Tn) corresponded to the sequence of discrete observation: practiced sport and (y0:Tn) was the sequence of continuous observations: steps, location distances, sleep time, app use, time at home, and location clusters count.

The dataset used in this study exhibited heterogeneity, encompassing both categorical variables such as practiced sports as well as continuous variables presumed to take real values (Table 1). For this purpose, we used the heterogenous-HMMs implementation from the HMMs in Python (PyHMM) library, which facilitates the use of various distributions to manage the emission probabilities of each feature type, as depicted in Figure 3 [34,55,57].

In this initial phase, the HMM was trained using 30% (296,072/986,909) of the available data. Once trained, the model was used to infer the state posterior probabilities. At each time step, the set of passive data for a given day and patient was represented by a hidden state posterior probability vector, with a total of 7 hidden states in this instance. Previous studies [34] tested different hidden state configurations, and 7 hidden components were found to effectively capture the underlying patterns in the data. The optimal number of hidden states was determined using the Bayesian Information Criterion and Akaike Information Criterion on a randomly selected subset of sequences with varying levels of missingness [58]. This number of hidden states also led to the best results when a classifier was applied to predict emotions.

The hidden states’ posterior probabilities refer to the probabilities of the hidden states given the observed data. These probabilities are calculated using the observations and the model parameters. The sequence of hidden states probability vectors served as the embedding of the daily information for each patient, enabling the training of subsequent models.

#### Sequence Forecasting With the Transformer Model

**Figure 4:**
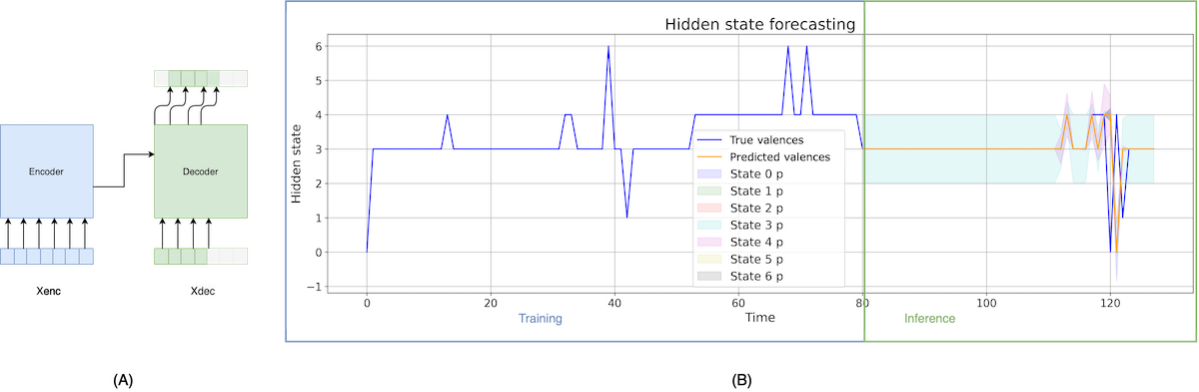
(A) Simplified encoder-decoder architecture of the transformer model. (B) Example of model training and inference. During training, the model learns the parameters to forecast the future state, using the real data as a reference. During inference, the transformer’s decoder is responsible for forecasting the future state value in an autoregressive manner. The blue line shows the true states, and the orange line shows those predicted by the model. During inference, the possible future states have associated probabilities, which are illustrated in the graph with different-colored margins around the orange line. Xenc: input sequence for the encoder; Xdec: input sequence for the decoder.

Due to the limited data on emotions and PHQ-9 responses, to obtain a more informative representation of the time series, we first performed a phase of self-supervised training, following a forecasting approach. To achieve this, we used a transformer model for time-series forecasting, leveraging its strengths in handling sequential data. Transformers have shown promising results in capturing long-range dependencies and can provide interpretability regarding the most relevant parts of the sequence for each specific task. Our transformer followed the basic encoder-decoder structure, as depicted in Figure 4 [10], incorporating elements from the encoder of the Informer model [12].

The encoder within the transformer architecture comprised a series of 3 layers. Each layer incorporated 2 distinct sublayers: a multihead self-attention mechanism and a position-wise fully connected feedforward network. Following each sublayer was a residual connection and layer normalization. Likewise, the decoder consisted of 3 layers. In addition to the 2 sublayers present in each encoder layer, the decoder incorporated a third sublayer responsible for performing cross-attention over the output generated by the encoder stack. Residual connections and layer normalization were similarly applied around each sublayer to the encoder. To prevent the decoder from attending to subsequent positions, modifications were made to the self-attention sublayer within the decoder stack, implementing causal (masked) self-attention.

Through the encoder, we obtained contextual information, and with the decoder, we performed the prediction of future observations, considering both the contextual information and the current observations. To incorporate information about the relative or absolute position of tokens in the time series, we combined a positional encoding with the input embeddings at the beginning of both the encoder and decoder stacks [10].

The transformer model was trained from scratch in a forecasting paradigm, wherein the transformer’s output was compared to the actual output shifted 1 day ahead. This approach enabled the model to predict future time points with precision. As our dataset consisted of real-world data, it was uncommon for the patients to have extensive sequences of data. Because of this limitation, for model training, we experimented with different sequence lengths, aiming to strike a balance between model performance and sequence duration. Through experimentation with various sequence lengths for both the encoder and decoder, we determined that a sufficiently large sequence (between 25 and 30 days) was required to adequately capture relationships and facilitate accurate forecasting.

For training, we used diverse training schedules, integrating early stopping and dropout techniques to mitigate data overfitting. The loss functions we tested included both mean squared error and mean absolute error, computed between the predicted and actual future sequences. Optimization was carried out using the Adam optimizer across 80 epochs.

A hyperparameter grid was defined, including values for embedding dimensions (32, 64, 128), which represent the dimension of the embedding vector for each data point; number of attention heads (4, 8, 16), indicating the number of heads in each attention layer; number of encoder and decoder layers (2, 3, 4, 6), referring to the attention blocks that include the attention, add and normalization, feedforward, and add and normalization layers; feedforward dimensions (128, 256, 512), which correspond to the dimension of the feedforward layers; dropout rate (0.3, 0.5); and learning rate (0.01, 0.001, 0.0005).

The best-performing model, which we presented in our results, was trained using sequences spanning 50 days of passive data, with 30 days allocated for the encoder and 20 days for the decoder. During inference, using the preceding 30 days of collected data was sufficient for forecasting, as the model could then predict further into the future autoregressively. The optimal model configuration was achieved with an embedding dimension of 32, 4 attention heads, 3 layers, a feedforward dimension of 128, a dropout rate of 0.3, and a learning rate of 0.001. The results obtained for the different combinations of hyperparameters, as well as a more detailed explanation of the used architecture, are included in Multimedia Appendix 2 [10,12,34,57].

#### Emotional State and PHQ-9 Score Classification

For this section, we used the output of the transformer model to train the subsequent classification layers, aimed at predicting specific targets. The objective was to predict emotional valence and the PHQ-9 scores for the following day.

For the prediction of emotional states, the HMM was first applied to the data sequences to obtain the posterior probabilities of the hidden states of the Markov model, which represented the embeddings of the passive data sequences. Subsequently, forecasting for the following days was conducted using the transformer, which was trained in the previous phase. These outputs were then used to train a model for emotional valence classification.

Several classification models were experimented with, including multilayer perceptron, ensemble models, support vector classifier, and extreme gradient boosting (XGB). Among these, random forest classifiers, as part of the ensemble models, and XGB demonstrated superior performance. This constituted the third training phase, which we evaluated through the *F*_1_-score, accuracy, precision-recall curves, and receiver operating characteristic (ROC) curves, along with their respective area under the curves (AUCs). For each input day, these models provided a probability distribution of the predicted emotional valence for the following day, which in this case was a probability distribution among 3 possible outcomes (0, 1, and 2). In this regard, we analyzed both the probability distributions of the valence predicted by the model and the concrete valence estimated for the following day (the one with the highest probability).

To predict the binary PHQ-9 score for each question, we compared several classifiers by training them with temporal windows of 15, 7, and 3 days using the transformer output (the contextual embeddings), the emotions predicted by the classifier, or a combination of the hidden states’ posterior probabilities and the predicted emotions.

### Ethical Considerations

Each clinical study received approval from the relevant institutional review board in compliance with ethical standards and the Declaration of Helsinki. Institutional review board approval numbers are indicated in brackets and correspond to the center where approval was obtained for each project and country. Patients at high risk of suicide were identified through collaborations with the Jiménez Díaz Foundation (FJD, EC005-21), Montpellier University Hospital (CPP Ouest IV 20/18_2), and Clínica Nuestra Señora de la Paz. Patients with CMDs were recruited from FJD (PIC148-22), while those with eating disorders were monitored at specialized mental health centers, including Adalmed and Ita mental health clinics. The study also includes patients with cancer monitored in partnership with Gregorio Marañón Hospital (EB2COLON2023), Centro Nacional de Investigaciones Oncológicas, and Fuenlabrada Hospital; patients with HIV/AIDS from Gregorio Marañón (MICRO.HGUGM.2022-002); patients with heart problems from Clínico San Carlos Hospital (19/239-O_P); and patients with obstructive sleep apnea monitored at FJD (PIC163-22). Informed consent was obtained from every participant at the time of inclusion, ensuring adherence to ethical guidelines and participant rights.

## Results

### Time-Series Forecasting

In this section, we first compare the time-series forecasting capabilities of the transformer and the HMM, without considering specific target predictions, solely as a comparison of their ability to capture the temporal structure and prediction performance of both models.

For the forecasting of the sequences of the posterior probabilities of the hidden states, which represent the information of the passive observations, our results demonstrated enhanced stability over time when comparing the transformer model to the HMM. Specifically, when both models performed autoregressive forecasting, predictions made by the transformer model exhibited greater similarity to the true values as we project further into the future beyond the last observed day.

**Figure 5:**
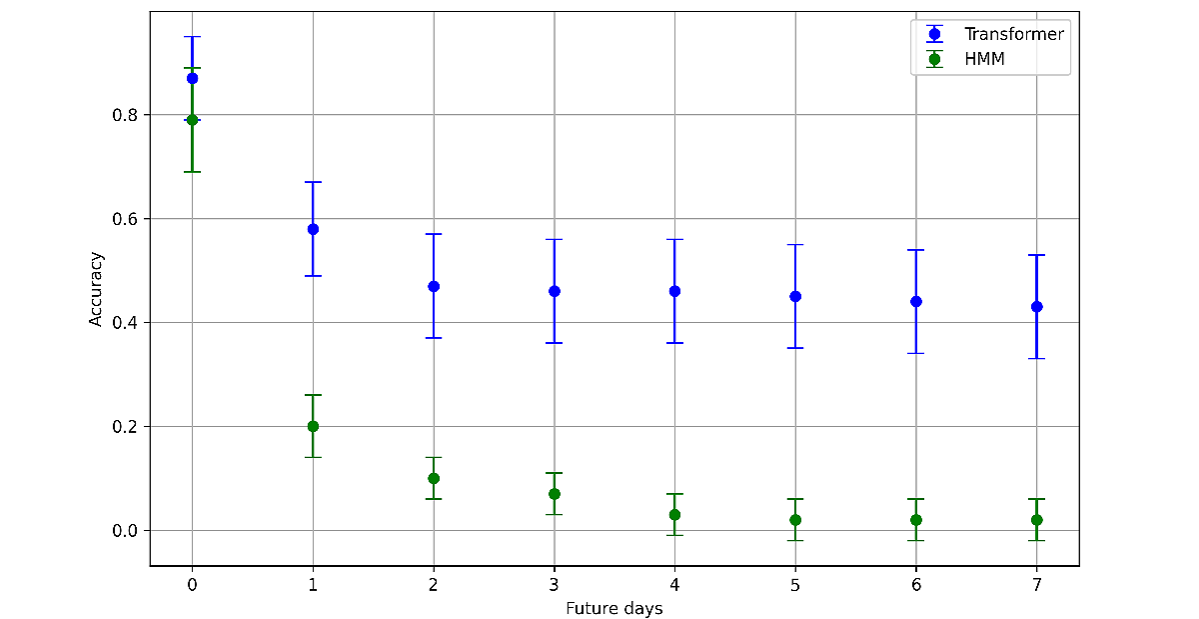
Comparison of models in state match forecasting. HMM: hidden Markov model.

Figure 5 presents the accuracy of state forecasting, measured as the rate of correctly predicted states for 0 to 7 days into the future. While both models performed similarly for immediate next-day predictions, the transformer’s predictions decayed more gradually than those of the HMM as the forecasting horizon extended. The chosen configuration for the transformer model involved training with sequences of 50 days—30 days for the encoder and 20 days for the decoder—during the initial training phase, encompassing all potential patient sequences. Training with shorter sequences yielded inferior forecasting performance.

### Emotional Valence Forecasting

Regarding emotional valence forecasting, our results focused on predicting emotional state 1 day ahead. The most effective approach involved using the XGB algorithm with a 7-day window of the transformer model decoder as input for the classification layers.

Alternative approaches were explored for the classification. One included incorporating model-embedded vectors of the hidden states, not the final output of the decoder, to potentially enhance informativeness; however, this yielded comparable results to only using the transformer’s decoder output. Another strategy involved retraining the entire model (including the transformer and classifier) or some of the layers via fine-tuning, as opposed to solely training the classifier while keeping the transformer parameters frozen to improve task performance. However, the results were similar, and given the time and resource requirements for complete retraining, we opted to train the classification layers for the specific task.

Results across different models used for classification exhibited minor variations, potentially attributed to the embedded vectors effectively capturing necessary information for forecasting, irrespective of the classification model’s complexity.

Table 2 shows the results for valence classification. Notably, the XGB model performed best with a ROC AUC of 0.982 and an accuracy of 0.93. Of particular interest was the discrimination of class number 2 (representing a neutral state), which is challenging to classify due to its scarcity. Despite a slight bias toward negative valence, models achieved robust discrimination for negative and positive states and notably good results for the neutral state, accurately predicting events 1 day in advance.

**Table 2:** Metrics for emotion classification models^a^.

Metric	MLP^b^	RF^c^	XGB^d^	XGB retrained	XGB embedding
Precision	0.86	0.87	0.89	*0.89*	0.84
Recall	0.82	*0.86*	*0.86*	0.85	0.8
*F*_1_-score	0.84	*0.87*	*0.87*	0.86	0.8
Accuracy	0.91	0.93	0.93	*0.94*	0.9
AUC^e^	0.97	0.98	*0.98*	0.98	0.92
PR^f^	0.89	0.92	*0.93*	0.9	0.88

^a^For each metric, the results obtained with the best model are italicized.

^b^MLP: multilayer perceptron.

^c^RF: random forest classifier.

^d^XGB: extreme gradient boosting.

^e^AUC: area under the curve.

^f^PR: precision-recall.

**Figure 6:**
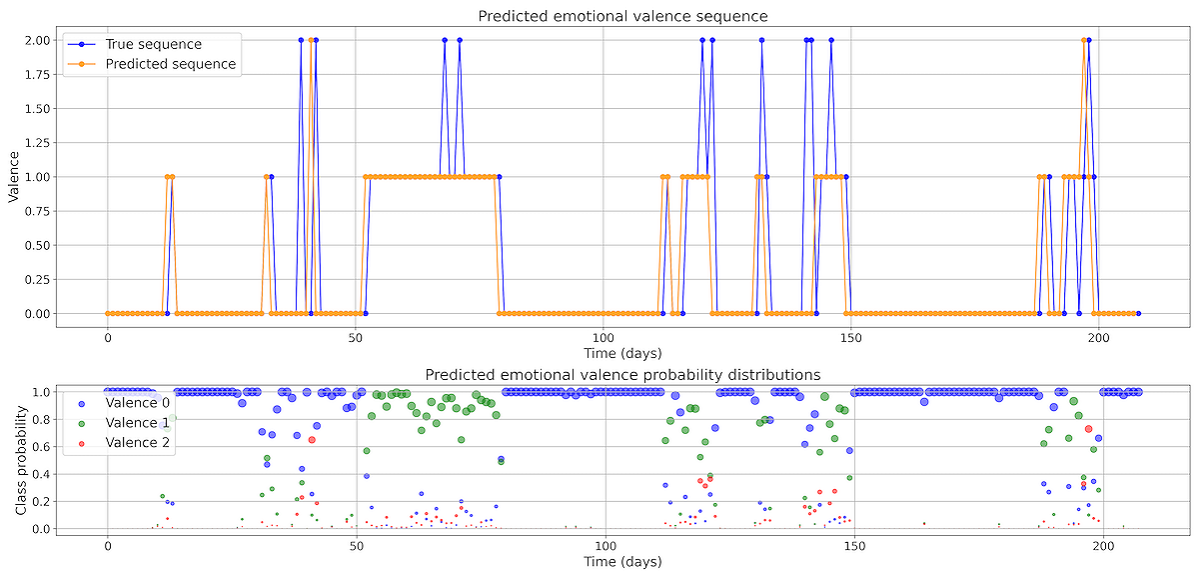
Sequences of real emotions (blue) and those predicted by the model (orange) for the following day. The colored circles denote the probability distribution of the emotional state. Purple indicates state 0 (negative valence), green indicates state 1 (neutral), and red indicates state 2 (positive valence). This figure pertains to a patient (ID: 218) selected from a set of patients with high variability of emotion in their temporal sequence.

Figure 6 illustrates individual patients’ emotional series, where the blue line represents the actual emotion for the current day, the orange line represents the forecasted emotion for the following day, and the circles represent the probabilities predicted by the classification model for each emotional valence for the next day. The position and size of the circles correspond to their probability values, with higher probabilities resulting in larger circles. Purple indicates valence 0 (negative), green indicates valence 1 (neutral), and red indicates valence 2 (positive).

Regarding emotional valence change detection, analyzing the emotional valence distributions revealed a pattern. During stable periods, the distributions were skewed, meaning that most of the probability mass was concentrated around a single valence outcome. However, near a change, the distributions become closer to a uniform distribution. That is, the probability mass was distributed more evenly among the possible outcomes.

To detect the change, we considered calculating the entropy and the Jensen-Shannon (JS) divergence within a temporal window of the sequence. We calculated the entropy of the valences’ probability distributions (equation 1) for a 3-day window, including the previous 3 days to each event. The JS divergence (equation 2) was computed between the present-day distribution and an average distribution over a temporal window to determine which approach could better determine the shift in emotional state: whether an increment in disorder within the window or a comparison between the actual and previous distribution.

H(x) = – Σ p_i_ log_2_ p_i_
**(1)**

D_JS_ (P||Q) = ½ Σ (p_i_ log_2_ p_i_/q_i_ + q_i_ log_2_ q_i_/p_i_) **(2)**

**Figure 7:**
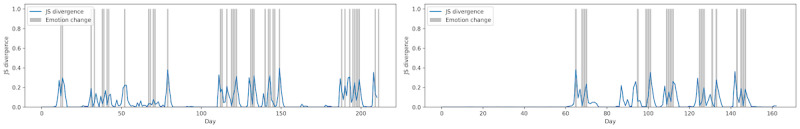
The Jensen-Shannon (JS) divergence calculated in a temporal window (blue curve) and the true emotion changes (gray vertical lines) for 2 patient sequences.

**Figure 8:**
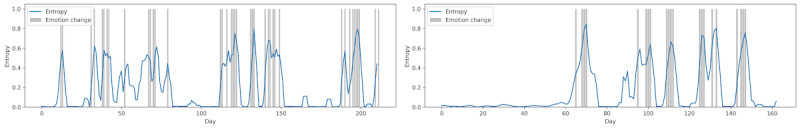
Entropy calculated in a temporal window (blue curve) and the true emotion changes (gray vertical lines) for 2 patient sequences.

In Figure 7, we display the real changes in emotional valence alongside the JS divergence, and in Figure 8, we display real changes and change detection through entropy. An increase in entropy within a 3-day temporal window, as seen in this case, is associated with a shift in emotional state. Similarly, divergence remains low, close to 0, during stable periods, with most peaks corresponding to real shifts in emotion.

**Figure 9:**
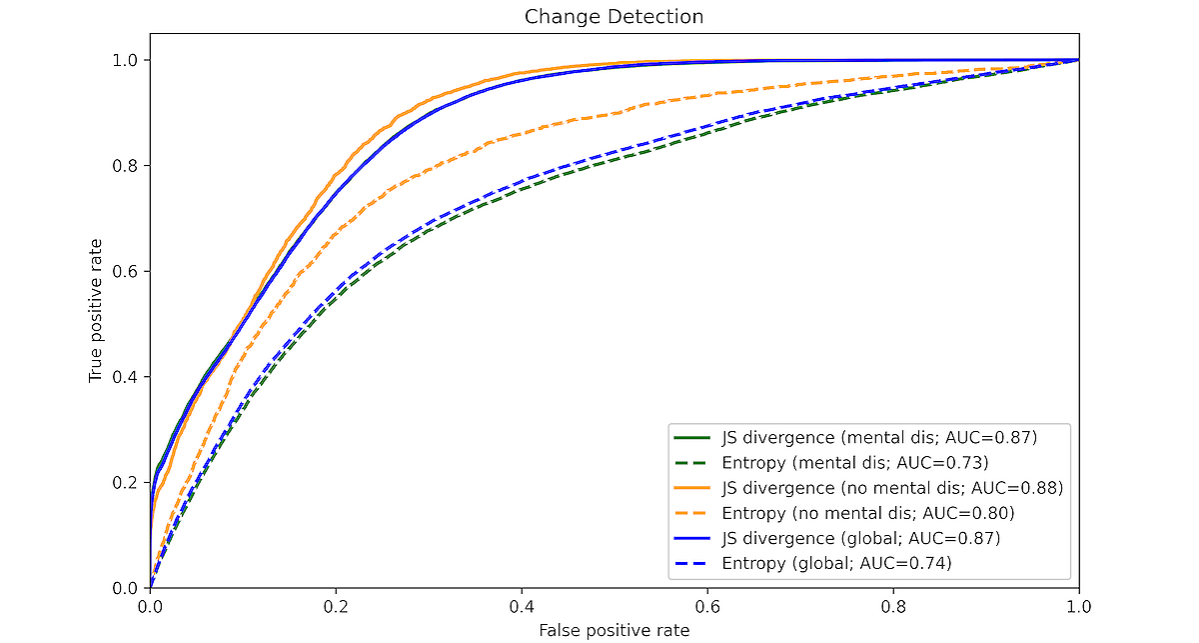
The receiver operating characteristic (ROC) curves for change detection with Jensen-Shannon (JS) divergence and entropy. The curves are obtained for change detection in patients with mental disorders, in patients without any diagnosed mental disorders, and for the entire study cohort (global). AUC: area under the curve; Mental dis.: patients with mental disorders; no mental dis.: patients without any diagnosed mental disorders.

Figure 9 shows the ROC AUC for emotional change detection with entropy and JS divergence. The results of change detection are shown for patients with mental disorders, for patients who have not been diagnosed with mental disorders, and for all study participants to compare detection across different scenarios. For the global case, lower thresholds for both metrics yielded better results, with JS divergence outperforming entropy by 0.13 ROC AUC for change detection.

### PHQ-9 Score Forecasting

Table 3 presents the accuracy and AUC results for the questionnaire score forecasting, delineated by the classification into 2 classes over a temporal window of 7 days using the decoder transformer outputs. The predictions were obtained using both a random forest classifier and an XGB classifier, both of which demonstrated similar results. Among the results presented in Table 3, the difference lies in the input provided to the classifier: either solely the transformer output (hidden states), the transformer output combined with the corresponding emotional state forecasting, or solely the emotional state. Notably, the results across all 3 configurations were similar, with slight improvements when using either the hidden states alone or solely the emotion distribution sequence as input.

When comparing different questions, certain inquiries demonstrated higher predictability than others. Notably, question 9 consistently yielded the most accurate predictions across all configurations. This question pertained to whether patients have experienced thoughts of being better off dead or hurting themselves. In addition, question 8, which addresses difficulties in movement or speech that others may notice, exhibited relatively accurate predictions, along with question 5, which was related to appetite, and question 7, which was related to concentration problems.

**Table 3:** Patient Health Questionnaire-9 (PHQ-9) score forecasting results for questions 1 to 9^a^.

PHQ-9 question	Transformer embeddings^b^			Transformer embeddings and emotion^c^			Emotion sequence (3 days)^d^			Emotion sequence (15 days)^d^	
	Accuracy	ROC^e^ AUC^f^	Accuracy		ROC AUC	Accuracy		ROC AUC	Accuracy		ROC AUC

1	0.57	0.62	0.57		0.60	*0.59* ^g^		*0.63*	0.57		0.61
2	0.6	0.60	0.57		0.59	*0.63*		*0.64*	0.63		0.63
3	0.55	0.51	0.55		0.50	0.55		0.55	*0.58*		*0.62*
4	*0.62*	*0.61*	0.53		0.49	0.53		0.54	0.57		0.57
5	0.54	0.57	0.55		0.60	*0.64*		*0.69*	0.53		0.60
6	0.46	0.46	0.47		0.51	0.6		0.51	0.61		0.65
7	0.52	0.54	0.55		0.54	0.61		0.62	*0.67*		*0.64*
8	0.64	0.52	0.61		0.53	0.68		0.50	*0.72*		*0.56*
9	*0.91*	*0.77*	0.91		0.74	0.91		0.60	0.91		0.74

^a^Results correspond to the classifier performance with different input sets. All the inputs correspond to sequences of different data types (as specified in the columns) before the day with a PHQ-9 record.

^b^Classifier inputs: sequence of embeddings obtained from the transformer.

^c^Classifier inputs: sequence of embeddings obtained from the transformer along with emotion valence prediction obtained from the emotion classifier.

^d^Classifier inputs: the inputs consist exclusively of the emotion predictions obtained from the emotion classifier. Therefore, these columns correspond to sequences of valence predictions of 3 and 15 days before the day with a PHQ-9 entry that is being predicted.

^e^ROC: receiver operating characteristic.

^f^AUC: area under the curve.

^g^For each question, the results obtained with the best model are italicized.

## Discussion

### Principal Findings

Regarding time-series forecasting, our findings indicated that the transformer model surpassed the HMM in predicting future time steps, resulting in more stable predictions. This suggests that attention mechanisms within the transformer model are effective in capturing longer temporal dependencies, leading to improved prediction stability. Such capabilities are particularly beneficial for assessing a patient’s state several days in advance, providing valuable insights into their potential behavior and enabling the early detection of high-risk situations.

The findings concerning emotional changes were generally positive, encompassing both global emotion detection results and change detection 1 day in advance. The variance in outcomes across different machine learning classification algorithms was minimal, highlighting the robustness of the variables and their latent representation. This indicated that accurately predicting a patient’s emotional state could be achieved solely through passive variables, with the representation of these variables in the posterior probabilities of the hidden states (obtained with generative models) proving to be informative.

When examining individual patient sequences, it became apparent that in many instances, we could correctly anticipate mood shifts in advance. As for change detection using measures such as entropy or divergence, there appeared to be a correlation between the disorder in emotional valence probability distribution within a specific temporal window and the subsequent emotional state change. Furthermore, global findings indicated that divergence yielded superior results for change detection, suggesting that identifying relative disorder between the current prediction distribution and the past-window distribution holds greater significance than overall disorder within the window, although both contribute to change detection. Similar results were obtained when comparing change detection in patients without any mental disorders and patients with mental disorders. This indicated that the detection was accurate in both cases, with slightly better results for the patients without mental disorders. This can be expected, as the literature suggests that these patients exhibit less fluctuation in their emotions compared to patients with mental disorders.

Regarding the PHQ-9 answers, our model aimed to predict responses with considerable accuracy across both high- and low-frequency classes, allowing for a comprehensive overview of the PHQ-9 outcomes. Upon analyzing differences between answers, those better predicted were typically the ones where patients found it easier to detect their emotions regarding the topic and the frequency of their thoughts. For instance, question 9, corresponding to “Thoughts that you would be better off dead, or of hurting yourself,” exhibited the highest classification AUC and accuracy. Conversely, answers such as “Trouble falling or staying asleep, or sleeping too much” and “Feeling bad about yourself or that you are a failure or have let yourself or your family down” yielded poorer results.

Having an approximate score on this questionnaire proved to be a valuable tool, facilitating the monitoring of changes in depressive symptoms over time and guiding treatment decisions. Consequently, it can be used for screening purposes to identify individuals who may require further evaluation for depression.

### Limitations

There was a high percentage of missing data in the passive variables, with the minimum percentage of missing data being 52.6% (519,168/986,909) for the number of steps and the maximum percentage being 69.47% (685,614/986,909) for time at home. In addition, there was a very high rate of missing data in the active variables we aimed to predict, such as emotional valence, which had 96.34% (950,833/986,909) missing data.

The recording of emotions was slightly imbalanced, with a higher rate of negative emotions recorded (negative: 16,536/36,076, 45.83%; positive: 10,972/36,076, 30.41%; and neutral: 8568/36,076, 23.74%). Consequently, there was greater sensitivity for detecting negative valence compared to neutral and positive states.

We had limited responses (n=549) for the PHQ-9, and they were sporadic for most of the patients. The mean interval between 2 responses was 25.27 days, with a mode of 14.5 days. On average, each patient responded 4.65 times. Thus, predicting the global score based on passive data was challenging.

### Conclusions

Our study yielded several key findings. First, the use of passive variables led to favorable outcomes in both emotion valence detection and change analysis. This underscores the potential of leveraging passive data sources for monitoring and understanding emotional states. Moreover, using temporal methods enabled accurate prediction of emotional states up to a day in advance, with stable results for subsequent days. This temporal stability in prediction highlights the robustness of our approach and suggests its potential applicability in real-world settings where timely intervention is crucial. Further exploration may elucidate the potential for extending prediction horizons beyond a single day, thereby enhancing the utility of our method in long-term monitoring scenarios. Furthermore, our model demonstrates promising performance in predicting PHQ-9 scores, providing an approximate understanding derived solely from passive data. This highlights the utility of this approach as a screening tool for identifying individuals at higher risk of having a crisis or whose depressive symptoms are changing, thereby enabling timely intervention or adjustment in treatment.

Future work should focus on the interpretability of the data. This includes exploring the impact of each variable to assess the patient’s condition as well as the effects of removing or adding new variables (such as heart rate and oxygen saturation). In addition, delving deeper into the understanding and extraction of information from the time series could help identify behavioral patterns that determine patient progression as well as pinpoint specific moments that are most relevant for changes in a patient’s emotional state, ultimately aiding in adapting the treatment.

While there is room for improvement, particularly in refining the predictive accuracy and expanding the scope of our analysis, our findings represent a significant step forward in the development of in situ support and unobtrusive monitoring strategies for mental health disorders.

## Appendix

Multimedia Appendix 1 Cohort overview and patient profiles.

Multimedia Appendix 2 Model description and implementation details.

## Abbreviations

AUC: area under the curve
BERT: Bidirectional Encoder Representations from Transformers
CMD: common mental disorder
eB2: Evidence-Based Behavior
EMA: ecological momentary assessment
HMM: hidden Markov model
JS: Jensen-Shannon
PHQ-9: Patient Health Questionnaire-9
PyHMM: hidden Markov models in Python
RoBERTa: Robustly Optimized Bidirectional Encoder Representations from Transformers Pretraining Approach
ROC: receiver operating characteristic
XGB: extreme gradient boosting
