# Supplementary material for: Emotion Forecasting: A Transformer-Based Approach
Source: J Med Internet Res. 2025 Mar 18;27:e63962. doi: 10.2196/63962 (PMC11962324; doi:10.2196/63962)
Supplement: Multimedia Appendix 1 [file jmir_v27i1e63962_app1.docx]

Data Appendix: Cohort Overview and Patient Profiles

The studies employing the eB2 platform span diverse patient populations and health conditions. Across all cohorts, the studies shared general inclusion criteria to ensure participants could effectively engage with the eB2 platform and related tools:

1. Participants had to be adults (aged 18 or older).
2. They were required to provide informed consent for participation.
3. They were required to own a compatible smartphone with Android (version 4.4 or higher) or iOS (version 10.0 or higher).

Exclusion criteria included:

1. Patients who did not frequently use their smartphones or were unable to operate mobile applications.
2. Refusal to sign the informed consent form.
3. Cognitive impairments or other conditions preventing understanding of the study requirements or the use of devices.

While these criteria were consistent across all cohorts, each study had its own specific eligibility requirements tailored to its research focus.

Below are detailed descriptions of the cohorts:

1. Common Mental Disorders (CMD): Conducted with Fundación Jiménez Díaz, this study focused on detecting physical, cognitive, and emotional changes in adults with CMD. Patients used the eB2 app to log emotions and complete the PHQ-9 questionnaire.
2. Early Neurodegenerative Disorders: In collaboration with IIS-FJD, this 4-month observational study assessed the feasibility of tools like the eB2 app, voice analysis, and gamification strategies in adults. Data collection included both passive monitoring and active assessments (WHOQOL-BREF, WHODAS, PHQ-9, GAD-7).
3. High Suicide Risk:
   1. Bipolar and Schizoaffective Disorders: Conducted at Clínica Nuestra Señora de la Paz, patients (ages 18-65) were monitored using the eB2 app, focusing on emotional modeling, predictive analysis, and the relationship between emotional scales and clinical data.
   2. Smart Crisis Project: At Fundación Jiménez Díaz and the University of Montpellier, 1000 patients in suicidal crisis were followed for six months using ecological momentary assessment (EMA) tools to explore links between sleep, appetite, and suicidal behaviors.
4. Cardiology Patients: At Hospital Clínico San Carlos, the study tracked physical recovery in adults undergoing major cardiac surgery, integrating socio-demographic and clinical data with eB2 monitoring. Exclusion criteria included low phone usage and cognitive impairments.
5. HIV Patients with Comorbidities: As part of the IntCare-CM project with Universidad Carlos III, 80 patients were monitored using wearable devices and AI tools to assess frailty and improve treatment adherence.
6. Colorectal Cancer: Conducted with Gregorio Marañón Hospital, this prospective study adapted the eB2 app for 131 patients with advanced/metastatic colorectal cancer, focusing on quality of life, diet, emotions, and functional status over one year.
7. Eating Disorders (TCA): In collaboration with ITA, studies targeted adolescents and young adults, requiring food intake reporting through the app, with optional emotional data logging.
