# Supplementary material for: Emotion Forecasting: A Transformer-Based Approach
Source: J Med Internet Res. 2025 Mar 18;27:e63962. doi: 10.2196/63962 (PMC11962324; doi:10.2196/63962)
Supplement: Multimedia Appendix 2 [file jmir_v27i1e63962_app2.docx]

Technical Appendix: Model Description and Implementation Details

Overview of Heterogeneous HMMs (HHMMs)

The HHMM extends traditional HMMs by combining continuous and discrete observations, enabling the modeling of heterogeneous data. Each hidden state is associated with two distinct probability distributions: (1) a Gaussian distribution for continuous observations and (2) a Multinoulli distribution for discrete observations [1]. This allows HHMMs to capture the conditional dependencies between hidden states and observations of different types. The key components include:

- Hidden States Sequence (S): Represents the latent state transitions.
- Continuous Observations (Y): Modeled by Gaussian emission probabilities.
- Discrete Observations (L): Modeled by multinomial emission probabilities.
- State Transition Probabilities (A): Define the likelihood of transitioning between states.
- Initial State Probabilities (π): Probabilities of the initial state.

HHMMs tackle the same three primary inference tasks:

1. Sequence likelihood estimation: given the observed data sequences {Y, L} and the model’s parameters θ = {A, B, D, π}, to estimate the probability of the observed sequence given the model: p (Y, L | θ).
2. Optimal hidden state sequence decoding: given {Y, L} and θ, to determine the optimal hidden states sequence S that better explains the data. This can be achieved by using the Forward-Backward algorithm, Yu and Kobayashi (2003), calculating p(sₜ | yₜ, lₜ) at each time-step, or through the Viterbi algorithm, Forney (1973), which maximizes the probability of the hidden states sequences by considering all time-steps t = {1, 2, ..., T}, i.e., calculating p(S | yₜ, lₜ).
3. To determine the optimal parameters θ that maximize the conditional probability p (Y, L | θ), which can be achieved through the Baum-Welch algorithm, Frazzoli (2013). The joint distribution required for this third task, which may be modified to support any other type of observation emission probability distribution, is expressed as:

$$p(S,Y,L)=\prod_{n=1}^{N} \left( p\left( s_{1}^{n} \right)\prod_{t=2}^{T_{n}} p\left( s_{t}^{n}|s_{t-1}^{n} \right) \right)\left( \prod_{t=1}^{T_{n}} p\left( y_{t}^{n}|s_{t}^{n} \right) \right)\left( \prod_{t=1}^{T_{n}} p\left( l_{t}^{n}|s_{t}^{n} \right) \right)$$

PyHHMM [1] offers several key functionalities that set it apart from other HMM implementations, such as hmmlearn, pomegranate, and pyro. While these libraries share basic features, PyHHMM includes unique capabilities tailored to address real-world data challenges:

1. Missing data inference: PyHHMM models can handle completely or partially missing observations during both training and inference.
2. Semi-supervised training: The model allows fixing discrete observation emission probabilities, which improves interpretability and accuracy by guiding the training process.
3. Model selection criteria: Implements Akaike Information Criterion (AIC) and Bayesian Information Criterion (BIC) to determine the optimal number of hidden states.
4. Flexible covariance matrices: Supports diagonal, full, tied, or spherical covariance matrices for Gaussian observations.
5. Synthetic data generation: Facilitates the simulation of sequential data from trained models.

Application in the Study

1. Model Choice: HHMMs were employed due to the dataset's mixed feature types, including real-valued (continuous) and categorical data.
2. Imputation: HMMs could be effectively used for imputing missing data due to their ability to marginalize over unobserved data using only the observed data. This is achieved through algorithms like Forward-Backward, which efficiently compute the probabilities of hidden states and observations, even in the presence of missing values. However, it is important to note that while this capability is one of the strengths of HMMs, in this study, they were not utilized as a method for data imputation.
3. Embedding Creation: Posterior probabilities of hidden states served as feature embeddings for downstream tasks, such as training transformer models.
4. State Selection: The optimal number of hidden states (7) was determined using BIC and AIC, ensuring effective pattern capture while balancing model complexity. Previous studies [2] tested different hidden state configurations, and 7 hidden components were found to effectively capture the underlying patterns in the data. To estimate the number of hidden states, the HMMs were trained on 91,047 sequences after filtering for those with at least 80% of observations in daily 48-slot sequences. The optimal number of hidden states was determined using the Bayesian Information Criterion (BIC) and Akaike Information Criterion (AIC) on a randomly selected subset of 10,000 sequences with varying levels of missingness. This number of hidden states also led to the best results when a classifier was applied to predict emotions and produce interpretable states.

Transformer Model Architecture Overview

The Transformer model used in this study follows the encoder-decoder architecture, as defined in the original Transformer model [3], with optimizations to enhance efficiency and scalability for time-series forecasting tasks. Below is a detailed description of the architecture:

Architecture

Encoder

The encoder processes the input sequence and generates an encoded sequence of hidden states. Each encoder layer consists of the following sub-layers:

1. Multi-Head Self-Attention: Each token in the input sequence attends to all other tokens, capturing relationships regardless of their distance in the sequence. This enables the model to efficiently capture contextual dependencies across the sequence. The self-attention mechanism is optimized through techniques like ProbSparse attention [4], which prioritizes the most significant query-key interactions, reducing computational complexity while maintaining performance.
2. Residual Connections: Each sub-layer (self-attention and feedforward) is followed by residual connections, helping to mitigate gradient vanishing issues and ensuring efficient training. These residual connections enable the gradients to flow more easily through the network, which improves convergence during training.
3. Feedforward Neural Network: After the self-attention operation, each token's representation passes through a fully connected (feedforward) layer, capturing complex features and patterns. This helps refine the token representations that have been contextualized through attention.
4. Layer Normalization: Each sub-layer (self-attention and feedforward) is followed by layer normalization, which stabilizes the training process by normalizing the outputs of each layer and reducing internal covariate shift.

The encoder is designed to process longer sequences by incorporating mechanisms that reduce the dimensionality of input features across layers while retaining the most important information. This hierarchical processing helps in managing long-range dependencies efficiently, allowing the encoder to learn more abstract representations at each successive layer.

Decoder

The decoder generates the output sequence by attending to both the encoder’s embedded sequence and the sequence input to the decoder. At each time step, the decoder only attends to the previous tokens in the sequence to predict the next token. Each decoder layer consists of the following components:

Each decoder layer includes:

1. Masked Multi-Head Self-Attention: In the decoder, the masked multi-head self-attention mechanism ensures autoregressive generation by preventing the decoder from attending to future tokens in the sequence. This maintains causal consistency during forecasting, meaning that predictions for future time steps depend only on past and current data. The self-attention layer is optimized with specific mechanisms like ProbSparse attention [4] which efficiently handles long sequences.
2. Residual Connections: Similar to the encoder, each sub-layer in the decoder (masked self-attention, cross-attention, and feedforward) includes residual connections. This ensures that the information flow is stable and efficient, allowing gradients to propagate smoothly through the network during training.
3. Cross-Attention: The cross-attention mechanism allows the decoder to attend to the encoder’s output, incorporating contextual information from the input sequence when generating the output. This enables the decoder to make predictions that are informed by both the input sequence and the previously generated tokens in the target sequence.
4. Feedforward Neural Network: After the attention mechanisms, each token's representation in the decoder passes through a feedforward network, which further refines the model's predictions and captures complex patterns between tokens.
5. Layer Normalization: Like the encoder, each sub-layer (masked self-attention, cross-attention, and feedforward) is followed by layer normalization, ensuring stable and efficient training.

Model Parameters

- Input Embedding Dimension: 64 dimensions, allowing the model to learn complex features for each input variable.
- Number of Attention Heads: 8 parallel attention mechanisms to capture diverse dependencies between tokens.
- Number of Encoder/Decoder Layers: 3 layers, where each encoder and decoder layer is the complete structure described earlier.
- Feedforward Dimension: 256 dimensions in the hidden layer of the feedforward networks, enabling the model to capture richer patterns.
- Dropout Rate: 0.5, to prevent overfitting and improve generalization.
- Input Dimension: 7 variables per time step.
- Output Dimension: 7 variables predicted per time step.
- Sequence Length: 50 time steps, allowing the model to consider temporal patterns over this range.

Model Training

The Transformer model was trained in a forecasting paradigm to predict future values based on past observations. Grid Search was used to explore different hyperparameter combinations, optimizing for the best model performance. During training, the Mean Squared Error (MSE) was used as the primary loss function and Mean Absolute Error (MAE) was also tracked for comparison.

The model was optimized using the Adam optimizer, known for its efficiency and adaptability to sparse gradients, which helps in faster convergence and better handling of large datasets. Additionally, Early Stopping was employed during training to prevent overfitting. Based on the Grid Search results, the model with the minimum MSE was selected for final evaluation and deployment.

Prediction Scenarios and Decoder Configurations

The model was trained to predict future sequences with two distinct scenarios.

#### Scenario 1: Forecasting of State Posterior Probabilities

In the first scenario, both the input and prediction of the decoder were represented as posterior probabilities of the hidden states. This setup allowed the model to predict the next set of probabilities, which were used for forecasting future sequences. Thus, in this case:

In the first scenario, both the input and the prediction of the decoder were sequences of posterior probabilities of the hidden states. Thus, in this case, the decoder provides a prediction of the posterior probability sequence for the following day.

1. Encoder input: Sequence of posterior probabilities of the hidden states (from the HHMM).
2. Decoder input: (Masked) sequence of posterior probabilities.
3. Decoder output: The decoder predicts a 7-dimensional probability vector for each day.

#### Scenario 2: Forecasting of Real Behavioral Data

In the second scenario, although the input to the encoder was still the posterior probabilities of the hidden states, the input and output of the decoder were the sequences of raw behavioral data (i.e., actual values of the data for each variable over time). In this scenario, the model handled missing data by masking missing values during training and computed the loss only for the observed data.

1. Encoder input: Posterior probabilities of the hidden states (from the HHMM).
2. Decoder input: Masked sequence of raw behavioral data (actual values for each variable), which might include missing values.
3. Decoder output: The decoder predicted the real behavioral data for each variable (e.g., number of steps, distance, etc), and the model learnt to forecast data based on the observed values.

In both prediction scenarios, Teacher Forcing was used during training to guide the model in predicting future steps more accurately. Teacher Forcing involves providing the actual observed data from the training set as the input to the decoder for the next prediction, instead of using the previous model's predictions. This helped the model learn more efficiently and avoided compounding prediction errors over time. Tables 1 and 2 show the results of the transformer’s training for both scenarios.

Autoregressive Prediction

During the autoregressive prediction phase, the model forecasted the data for the following day.

1. In Scenario 1, the decoder's output was a probability vector for each day, representing the predicted state’s probability of future values.
2. In Scenario 2, the output of the decoder was a vector of predicted real data values (e.g., number steps, distance, etc.).

This distinction allows the model to handle both probabilistic and raw data prediction tasks, depending on the specific scenario. In this case, during the autoregressive phase, teacher forcing was no longer employed. Instead, the predicted output of the decoder for the previous day was used as the input to predict the following day. Consequently, this approach can lead to a gradual degradation in the model's performance over time.

Error measures (MSE and MAE) were used to identify the best hyperparameter combination in the forecasting task for each scenario, so they were each computed with different types of data in each of them. For an objective comparison of both approaches related to the specific task following the sequence forecasting, both were compared based on their performance in the emotional valence classification. Table 3 shows the results for the valence classification for both approaches.

From this comparison, it was observed that the model that achieved the best classification was the one utilizing the probability sequences. This model was ultimately chosen, with the hyperparameter combination shown in the first row of Table 1.

Table 1. Result of MSE and MAE for the 20 hyperparameter combinations with the best results for probability sequence forecasting.

| Dim  model | Number of head | Number of layers | Dim feedforward | Dropout | Lr | MSE | Std MSE | MAE | Std MAE |
| --- | --- | --- | --- | --- | --- | --- | --- | --- | --- |
| 32.000 | 4.000 | 3.000 | 128.000 | 0.300 | 0.001 | 3.371 | 12.966 | 0.743 | 1.679 |
| 32.000 | 8.000 | 4.000 | 128.000 | 0.300 | 0.001 | 3.374 | 12.968 | 0.735 | 1.684 |
| 32.000 | 4.000 | 3.000 | 256.000 | 0.300 | 0.001 | 3.381 | 12.942 | 0.734 | 1.686 |
| 32.000 | 16.000 | 3.000 | 128.000 | 0.300 | 0.001 | 3.384 | 12.959 | 0.732 | 1.687 |
| 32.000 | 8.000 | 3.000 | 512.000 | 0.300 | 0.001 | 3.397 | 12.976 | 0.730 | 1.692 |
| 32.000 | 4.000 | 4.000 | 512.000 | 0.300 | 0.001 | 3.397 | 12.923 | 0.744 | 1.686 |
| 32.000 | 8.000 | 4.000 | 256.000 | 0.300 | 0.001 | 3.405 | 13.087 | 0.717 | 1.700 |
| 32.000 | 4.000 | 4.000 | 128.000 | 0.300 | 0.001 | 3.413 | 12.951 | 0.761 | 1.683 |
| 32.000 | 8.000 | 6.000 | 128.000 | 0.300 | 0.001 | 3.413 | 12.958 | 0.744 | 1.691 |
| 32.000 | 16.000 | 3.000 | 256.000 | 0.300 | 0.001 | 3.416 | 13.052 | 0.735 | 1.696 |
| 32.000 | 4.000 | 4.000 | 256.000 | 0.300 | 0.001 | 3.418 | 12.970 | 0.750 | 1.690 |
| 32.000 | 4.000 | 6.000 | 128.000 | 0.300 | 0.001 | 3.422 | 13.128 | 0.721 | 1.703 |
| 32.000 | 16.000 | 4.000 | 256.000 | 0.300 | 0.001 | 3.422 | 13.076 | 0.733 | 1.699 |
| 32.000 | 8.000 | 3.000 | 128.000 | 0.300 | 0.001 | 3.445 | 13.119 | 0.760 | 1.693 |
| 32.000 | 8.000 | 6.000 | 256.000 | 0.300 | 0.001 | 3.455 | 13.117 | 0.723 | 1.712 |
| 32.000 | 16.000 | 4.000 | 128.000 | 0.300 | 0.001 | 3.462 | 13.166 | 0.763 | 1.697 |
| 32.000 | 4.000 | 6.000 | 256.000 | 0.300 | 0.001 | 3.484 | 13.268 | 0.754 | 1.708 |
| 32.000 | 8.000 | 3.000 | 256.000 | 0.300 | 0.001 | 3.493 | 13.110 | 0.756 | 1.709 |
| 32.000 | 16.000 | 6.000 | 256.000 | 0.300 | 0.001 | 3.501 | 13.069 | 0.767 | 1.707 |
| 32.000 | 4.000 | 3.000 | 512.000 | 0.300 | 0.001 | 3.517 | 13.376 | 0.751 | 1.718 |

Table 2. Result of MSE and MAE for the 20 hyperparameter combinations with the best results for behavioral sequence forecasting.

| Dim  model | Number of head | Number of layers | Dim feedforward | Dropout | Lr | MSE | Std MSE | MAE | Std MAE |
| --- | --- | --- | --- | --- | --- | --- | --- | --- | --- |
| 32.000 | 16.000 | 6.000 | 256.000 | 0.300 | 0.001 | 0.793 | 2.748 | 0.561 | 0.692 |
| 64.000 | 8.000 | 3.000 | 512.000 | 0.300 | 0.001 | 0.814 | 2.886 | 0.552 | 0.713 |
| 64.000 | 4.000 | 3.000 | 128.000 | 0.300 | 0.001 | 0.817 | 2.886 | 0.560 | 0.709 |
| 32.000 | 8.000 | 4.000 | 512.000 | 0.300 | 0.001 | 0.820 | 2.867 | 0.558 | 0.713 |
| 64.000 | 8.000 | 3.000 | 128.000 | 0.300 | 0.001 | 0.821 | 2.864 | 0.566 | 0.708 |
| 32.000 | 16.000 | 4.000 | 256.000 | 0.300 | 0.001 | 0.823 | 2.889 | 0.564 | 0.710 |
| 32.000 | 16.000 | 4.000 | 512.000 | 0.300 | 0.001 | 0.826 | 2.852 | 0.571 | 0.707 |
| 32.000 | 8.000 | 4.000 | 128.000 | 0.300 | 0.001 | 0.827 | 2.909 | 0.562 | 0.714 |
| 32.000 | 8.000 | 4.000 | 512.000 | 0.500 | 0.001 | 0.828 | 2.806 | 0.573 | 0.706 |
| 64.000 | 16.000 | 3.000 | 512.000 | 0.300 | 0.001 | 0.828 | 2.863 | 0.566 | 0.712 |
| 64.000 | 16.000 | 4.000 | 256.000 | 0.300 | 0.001 | 0.829 | 2.886 | 0.560 | 0.718 |
| 32.000 | 4.000 | 3.000 | 128.000 | 0.300 | 0.001 | 0.829 | 2.867 | 0.567 | 0.713 |
| 32.000 | 4.000 | 6.000 | 128.000 | 0.300 | 0.001 | 0.830 | 2.936 | 0.562 | 0.717 |
| 32.000 | 8.000 | 6.000 | 128.000 | 0.300 | 0.001 | 0.830 | 2.864 | 0.569 | 0.711 |
| 64.000 | 16.000 | 3.000 | 256.000 | 0.300 | 0.001 | 0.830 | 2.961 | 0.560 | 0.719 |
| 64.000 | 4.000 | 4.000 | 256.000 | 0.300 | 0.001 | 0.832 | 2.930 | 0.567 | 0.714 |
| 64.000 | 4.000 | 4.000 | 512.000 | 0.300 | 0.001 | 0.833 | 2.918 | 0.558 | 0.722 |
| 64.000 | 4.000 | 4.000 | 128.000 | 0.500 | 0.001 | 0.833 | 2.907 | 0.565 | 0.717 |
| 32.000 | 16.000 | 3.000 | 512.000 | 0.300 | 0.001 | 0.834 | 2.855 | 0.570 | 0.714 |
| 32.000 | 8.000 | 4.000 | 256.000 | 0.300 | 0.001 | 0.834 | 2.896 | 0.574 | 0.711 |

Table 3. Results for valence classification with sequences of posterior probabilities and behavioral data.

|  | Hidden states posterior probabilities | Behavioral data |
| --- | --- | --- |
| Precision | 0.89 | 0.86 |
| Recall | *0.86* | *0.84* |
| F1-Score | *0.87* | *0.85* |
| Accuracy | 0.93 | 0.92 |
| AUC | *0.98* | 0.95 |

References

1. Moreno-Pino F, Sükei E, Olmos PM, Artés-Rodríguez A. PyHHMM: A Python Library for Heterogeneous Hidden Markov Models. Preprint. arXiv. 2022;2201.06968. Available at: <http://arxiv.org/abs/2201.06968>.
2. Sükei E, Norbury A, Perez-Rodriguez M, Olmos P, Artés A. Predicting Emotional States Using Behavioral Markers Derived from Passively Sensed Data: Data-Driven Machine Learning Approach. JMIR Mhealth Uhealth. 2021;9(3):e24465. doi:10.2196/24465.
3. Vaswani A, Shazeer N, Parmar N, Uszkoreit J, Jones L, Gomez AN, Kaiser L, Polosukhin I. Attention Is All You Need. arXiv. 2017 Jun. arXiv:1706.03762. doi: 10.48550/arXiv.1706.03762.
4. Zhou, Haoyi, et al. "Informer: Beyond efficient transformer for long sequence time-series forecasting." Proceedings of the AAAI conference on artificial intelligence. Vol. 35. No. 12. 2021.
